# The derivative-free Fourier shell identity for photoacoustics

**DOI:** 10.1186/s40064-016-3294-y

**Published:** 2016-09-19

**Authors:** Natalie Baddour

**Affiliations:** Department of Mechanical Engineering, University of Ottawa, 161 Louis Pasteur, Ottawa, K1N 6N5 Canada

**Keywords:** Photoacoustics, Frequency domain photoacoustics, Mathematical physics, Tomography, Fourier-based tomography

## Abstract

In X-ray tomography, the Fourier slice theorem provides a relationship between the Fourier components of the object being imaged and the measured projection data. The Fourier slice theorem is the basis for X-ray Fourier-based tomographic inversion techniques. A similar relationship, referred to as the ‘Fourier shell identity’ has been previously derived for photoacoustic applications. However, this identity relates the pressure wavefield data function and its normal derivative measured on an arbitrary enclosing aperture to the three-dimensional Fourier transform of the enclosed object evaluated on a sphere. Since the normal derivative of pressure is not normally measured, the applicability of the formulation is limited in this form. In this paper, alternative derivations of the Fourier shell identity in 1D, 2D polar and 3D spherical polar coordinates are presented. The presented formulations do not require the normal derivative of pressure, thereby lending the formulas directly adaptable for Fourier based absorber reconstructions.

## Background

Photoacoustics is the generation of acoustic waves as a consequence of the absorption of light energy by an absorbing material and the subsequent thermo-elastic expansion of the material. The method combines the spatial resolution of ultrasound with the contrast of optical absorption for deep imaging in biological tissues (Xu and Wang 2006; Telenkov et al. 2009; Telenkov and Mandelis 2009), and has therefore shown great promise for biomedical imaging applications.

In X-ray tomography, the Fourier slice theorem provides a relationship between the Fourier components of the object being imaged and the measured projection data (Slaney and Kak 1988) and is the basis for Fourier-based inversion techniques (Chandra et al. 2014). A similar relationship for Photoacoustic Tomography (PAT) would also be very useful. Anastasio et al. (2007) derived the “Fourier shell identity”, a mathematical relationship between the pressure wavefield data function, its normal derivative measured on an arbitrary aperture that encloses the object and the three-dimensional (3D) Fourier transform of the optical absorption distribution evaluated on concentric spheres. This relationship can be regarded as the PAT analog of the classic Fourier slice theorem from X-ray tomography. It provides a mapping that relates the temporal Fourier component of the pressure data and its normal derivative to a specified spatial Fourier component of the source object function. The difficulty with Anastasio et al’s derivation is that knowledge of the normal derivative of pressure is required—a value that is not normally obtained through experiments.

In this paper, the Fourier shell identity in 1, 2 and 3 dimensions, such that the normal derivative is not required, are derived and presented. This provides a direct relationship between the temporal Fourier components of the pressure data and the Fourier components of the object function. The Fourier shell identity as derived in this paper requires no additional information than that typically acquired in experiments and thus can be directly used for absorber reconstructions.

## Photoacoustic governing equations

Diebold gives a concise explanation of the governing equation for the pressure that results from launching a photoacoustic wave (Diebold 2009). The governing equation is given by

1 $$ \left[ {\nabla^{2} - \frac{1}{{c_{s}^{2} }}\frac{{\partial^{2} }}{{\partial t^{2} }}} \right]p\left( {\vec{r},t} \right) = - \frac{\beta }{{C_{p} }}\frac{{\partial H\left( {\vec{r},t} \right)}}{\partial t} $$

where $\beta$ is the thermal expansion coefficient, $c_{s}$ is the speed of sound, $C_{p}$ is the specific heat, *H* is the energy per unit volume and time deposited by the optical radiation beam, and $p\left( {\vec{r},t} \right)$ is the pressure of the acoustic wave, a function of space ($\vec{r}$) and time, *t*. As is common, it is assumed that *H* is a separable function of space and time, so that $H\left( {\vec{r},t} \right) = A\left( {\vec{r}} \right)I(t)$. In this work, the temporal Fourier transform is employed with the angular frequency, non-unitary convention for forward and inverse transforms, as given by

2 $$ \tilde{f}(\omega ) = \int\limits_{ - \infty }^{\infty } {f(t)e^{ - i\omega t} dt} \;\;\;\; \Leftrightarrow \;\;\;f\left( t \right) = \frac{1}{2\pi }\int\limits_{ - \infty }^{\infty } {\tilde{f}\left( \omega \right)e^{i\omega t} d\omega } \; $$

where a tilde ($\sim$) over the variable has been used to denote a temporal Fourier transform, and assuming suitability of the function *f* for Fourier transformation. The same formulation can be used to define spatial Fourier transforms, where an overhat notation is used to denote a spatial Fourier transform where the spatial variable $\vec{r}$ is transformed to the spatial frequency variable $\vec{\omega }$, so for example $f\left( {\vec{r}} \right)$ is spatially Fourier transformed to $\hat{f}\left( {\vec{\omega }} \right)$. Taking the temporal Fourier transform of (1), it then follows that

3 $$ \nabla^{2} \tilde{p}\left( {\vec{r},k} \right) + k^{2} \tilde{p}\left( {\vec{r},k} \right) = - \frac{{ik\beta c_{s} }}{{C_{p} }}\tilde{I}\left( k \right)A\left( {\vec{r}} \right) $$

where $k = \omega /c_{s}$ is the wave number.

## Fourier shell identity

Anastasio et al. (2007) demonstrated a mathematical relationship between the pressure wavefield data function and its normal derivative measured on an arbitrary aperture that encloses the object and the three-dimensional (3D) Fourier transform of the optical absorption distribution evaluated on concentric spheres. They referred this relationship as a ‘Fourier-shell identity’, analogous to the well-known Fourier slice theorem of X-ray tomography. The Fourier-shell identity as derived by Anastasio et al. (2007) is given by

4 $$ \hat{A}\left( {\vec{\omega } = k{\hat{\mathbf{s}}}} \right) = \frac{{iC_{p} }}{{kc_{s} \beta \tilde{I}\left( k \right)}}\int_{{\varOmega_{0} }} {\left[ {{\mathbf{\hat{n}^{\prime}}} \cdot \nabla \tilde{p}\left( {\vec{r}_{0}^{\prime } ,k} \right) + ik{\mathbf{\hat{n}^{\prime}}} \cdot {\hat{\mathbf{s}}}\;\tilde{p}\left( {\vec{r}_{0}^{\prime } ,k} \right)} \right]} \;e^{{ - ik{\hat{\mathbf{s}}} \cdot \vec{r}_{0}^{\prime } }}\,dS^{\prime} $$

where $\varOmega_{0}$ is an arbitrary measurement surface that is smooth, closed and encloses the object, $dS^{\prime}$ is the differential surface element on $\varOmega_{0}$, ${\mathbf{\hat{n}^{\prime}}}$ is the outward normal vector to $\varOmega_{0}$ at the point $\vec{r}_{0}^{\prime } \in \varOmega_{0}$, ${\hat{\mathbf{s}}}$ is an arbitrary unit vector and the $\hat{A}\left( {\vec{\omega }} \right)$ indicates the 3D spatial Fourier transform of $A\left( {\vec{r}} \right)$.

In practical applications, the normal derivative of the pressure wavefield will not be measured so the Fourier shell identity as given in (4) is not immediately useful. Equation (4) permits determination of concentric ‘shells’ of Fourier components of $\hat{A}\left( {\vec{\omega } = k{\hat{\mathbf{s}}}} \right)$ from knowledge of $\tilde{p}\left( {\vec{r}_{0}^{\prime } ,k} \right)$ and its derivative along the ${\mathbf{\hat{n}^{\prime}}}$. Because ${\hat{\mathbf{s}}}$ can be chosen to specify any direction, $\hat{A}\left( {\vec{\omega } = k{\hat{\mathbf{s}}}} \right)$ specifies the Fourier components of $\hat{A}\left( {\vec{\omega }} \right)$ that reside on a spherical surface of radius |k|, whose center is at the origin.

It is shown in the proceeding sections how Eq. (4) can be made specific for the 1D, 2D polar and 3D spherical polar cases. The identity is also re-derived in those three cases so that the derivative of pressure does not appear in the formulation.

## Analysis in 1D

### Fourier shell identity in 1D

Using the same approach as used for the original derivation in Anastasio et al. (2007), it is shown in the “Appendix” that the 1D expression of the Fourier shell identity is given by

5 $$ \hat{A}\left( k \right) = \frac{{iC_{p} }}{{k\beta c_{s} \tilde{I}\left( k \right)}}\left[ {e^{{ - ikz_{0} }} \left( {\frac{d}{dz}\tilde{p}\left( {z_{0} ,k} \right) + ik\tilde{p}(z_{0} ,k)} \right) - e^{{ikz_{0} }} \left( {\frac{d}{dz}\tilde{p}\left( { - z_{0} ,k} \right) + ik\tilde{p}( - z_{0} ,k)} \right)} \right] $$

Equation (5) is the 1D equivalent of Eq. (4), the Fourier shell identity. In Eq. (5), the overhat indicates a spatial Fourier transform in the 1D spatial variable, *z*, meaning that the spatial variable *z* transforms to the spatial frequency variable $\omega_{z}$. Equation (5) at first appears different from Anastasio’s formulation in Eq. (4). The source of this apparent difference is that in it is necessary to enclose the source function and to do so in 1D, the “detecting surface” must be located at *both*$z = \pm z_{0}$. This follows because a detector at only one of $z = \pm z_{0}$ will not enclose the source.

### Multivariable Fourier analysis

To proceed with the derivation in this manuscript, the photoacoustic Eq. (1) is considered in one dimensional Cartesian coordinates so that position is a function of *z* only, $\vec{r} = \left( z \right)$. Taking the temporal Fourier transform of (1) (denoted with an over-tilde) so that time, *t*, transforms to the temporal frequency variable $\omega$, and then further taking a spatial Fourier transform (denoted with an overhat) in the spatial variable, *z*, so that spatial variable *z* transforms to the spatial frequency variable $\omega_{z}$, leads to

6 $$ \hat{\tilde{p}}\left( {\omega_{z} ,\omega } \right) = \frac{{ik\beta c_{s} }}{{C_{p} }}\,\tilde{I}\left( \omega \right)\,\frac{{\hat{A}\left( {\omega_{z} } \right)}}{{\omega_{z}^{2} - k^{2} }} $$

where $k = \omega /c_{s}$ is the wave number.

### The Fourier shell theorem in 1D

From (6), the inverse spatial Fourier transform can be computed with the help of the appropriate choice of Theorem 5 from Baddour (2011). The third option of Theorem 5 from Baddour (2011) states that the following result holds true:

7 $$ \frac{1}{2\pi }\int\limits_{ - \infty }^{\infty } {\frac{\phi (\rho )}{{\rho^{2} - k^{2} }}} e^{i\rho z} \;d\rho = \left\{ {\begin{array}{*{20}l} {\frac{1}{2ik}\phi \left( { - k} \right)e^{ - ikz} } \hfill &\quad {z > 0} \hfill \\ {\frac{1}{2ik}\phi \left( k \right)e^{ikz} } \hfill &\quad {z < 0} \hfill \\ \end{array} } \right. $$

*Assuming that*$\hat{A}\left( {\omega_{z} } \right)$*has no poles* and remains bounded, then the application of the inverse spatial Fourier transform (2) to (6), while making use of the identity in (7) gives

8 $$ \begin{aligned} \tilde{p}\left( {z,\omega } \right) & = \frac{1}{2\pi }\int\limits_{ - \infty }^{\infty } {\hat{\tilde{p}}\left( {\omega_{z} ,\omega } \right)e^{{i\omega_{z} z}} d\omega_{z} } = \frac{{\beta c_{s} ik}}{{2\pi C_{p} }}\tilde{I}\left( \omega \right)\int\limits_{ - \infty }^{\infty } {\frac{{\hat{A}\left( {\omega_{z} } \right)}}{{\omega_{z}^{2} - k^{2} }}e^{{i\omega_{z} z}} d\omega_{z} } \\ & = \frac{{\beta c_{s} }}{{2C_{p} }}\tilde{I}\left( \omega \right)\left\{ {\begin{array}{*{20}l} {\hat{A}\left( { - k} \right)e^{ - ikz} } \hfill &\quad {z > 0} \hfill \\ {\hat{A}\left( k \right)e^{ikz} } \hfill &\quad {z < 0} \hfill \\ \end{array} } \right. \\ \end{aligned} $$

Equation (8) is the statement of the Fourier shell identity in 1D. This result is very powerful as it relates the temporal Fourier components of pressure $\tilde{p}\left( {z,\omega } \right)$ directly to $\hat{A}\left( k \right)$, which represents the Fourier components of the spatial Fourier transform of the absorber evaluated at $\omega_{z} = k$ where $k = \omega /c_{s}$. It is shown in the next section that this is exactly the same as the result derived by Anastasio et al. (2007) for the Fourier shell identity, however Eq. (8) does not require knowledge of the derivative of pressure and thus can be easily inverted. For example, Eq. (8) implies measurements made at some detector location $z = z_{0} < 0$ (measurement in reflection) can be used to write

9 $$ \hat{A}\left( {\frac{\omega }{{c_{s} }}} \right) = \frac{{2C_{p} \tilde{p}\left( {z_{0} ,\omega } \right)}}{{\beta c_{s} \tilde{I}\left( \omega \right)}}e^{{ - i\frac{{z_{0} }}{{c_{s} }}\omega }} $$

Hence, the success in being able to reconstruct $A\left( z \right)$ is partly in the ability to gather sufficient (*k* intensive) information about $\hat{A}\left( k \right)$ to enable the inverse spatial Fourier transform to be computed.

Assuming $z = z_{0} < 0$ (which corresponds to a measurement in reflection) and using (9), then

10 $$ A\left( z \right) = \frac{1}{2\pi }\int\limits_{ - \infty }^{\infty } {\hat{A}(k)e^{ikz} dk} = \frac{{C_{p} }}{{\pi \beta c_{s}^{2} }}\int\limits_{ - \infty }^{\infty } {\frac{{\tilde{p}\left( {z_{0} ,\omega } \right)}}{{\tilde{I}\left( \omega \right)}}e^{{i\frac{{\omega \left( {z - z_{0} } \right)}}{{c_{s} }}}} d\omega } $$

Equation (10) requires measurements of $\tilde{p}\left( {z_{0} ,\omega } \right)$ and $\tilde{I}\left( \omega \right)$ at a sufficiently wide bandwidth to enable the Fourier integral to be computed or approximated. In Eq. (10), the $z_{0} /c_{s}$ term in the exponential represents the time taken for the signal to travel from the absorber to the detector and that time is an indication of the spatial location of the absorber. The term in front of the integral is simply a scaling term. Therefore, Eq. (10) states that the absorber profile in space can be found by finding the inverse temporal Fourier transform $\bar{A}\left( t \right) = {\mathbb{F}}^{ - 1} \left\{ {\tilde{p}\left( {z_{0} ,\omega } \right)/\tilde{I}\left( \omega \right)|\omega \to t} \right\}$ via well known numerical techniques for calculating Fourier transforms and then scaling $A\left( z \right) = \bar{A}\left( {c_{s} t} \right)$ to find the shape of the absorber function as a function of space.

### Comparison with the Fourier shell identity

Now, Eq. (5) is the “Fourier shell identity” as put forward by Anastasio et al. (2007), where the temporal Fourier component of pressure values $\tilde{p}(z_{0} ,k)$ and its derivative are related to the values of a specified Fourier component, $\hat{A}\left( k \right)$. The problem with the formulation in (5) is the requirement for measurements of the derivative of $\tilde{p}(z_{0} ,k)$, which does not occur in practice. However, it can be shown that the formulation presented in this paper, as given in Eq. (8), leads to the same result in Eq. (5).

Using the newly derived form of the Fourier shell theorem as given in Eq. (8), evaluate

11 $$ \left. {\frac{{iC_{p} }}{{k\beta c_{s} \tilde{I}\left( k \right)}}\left\{ {e^{ - ikz} \frac{d}{dz}\tilde{p}\left( {z,k} \right) - \tilde{p}(z,k)\left. {\frac{d}{dz}e^{ - ikz} } \right|_{{ - z_{0} }}^{{z_{0} }} } \right\}} \right|_{{\tilde{p}\left( {z,k} \right)\,=\,\frac{{\beta c_{s} }}{{2C_{p} }}\tilde{I}\left( k \right)\left\{ {\begin{array}{*{20}l} {\hat{A}\left( { - k} \right)e^{ - ikz} } \hfill &\quad {z > 0} \hfill \\ {\hat{A}\left( k \right)e^{ikz} } \hfill &\quad {z < 0} \hfill \\ \end{array} } \right.}} $$

Simplifying (11) gives

12 $$ \frac{{iC_{p} }}{{k\beta c_{s} \tilde{I}\left( k \right)}}\left\{ { - \frac{{ik\beta c_{s} }}{{C_{p} }}\tilde{I}\left( k \right)\hat{A}\left( k \right)} \right\} = \hat{A}\left( k \right) $$

Hence, the version of the Fourier shell theorem in 1D as presented in Eq. (8) satisfies the Fourier shell identity in 1D as derived by Anastasio et al. (2007) and shown in Eq. (5). However, the formulation of Eq. (8) provides a useful alternative since no knowledge or measurement of the derivative of pressure is required.

## Analysis in 2D polar coordinates

### 2D polar coordinates

For a 2D function in polar coordinates, the function can be written as $f\left( {\vec{r}} \right) = f(r,\theta )$ and the $\theta$ dependence can be expanded into a Fourier series due to the $2\pi$ periodicity of the function in $\theta$. Hence,

13 $$ f(r,\theta ) = \sum\limits_{n = - \infty }^{\infty } {f_{n} (r)e^{in\theta } } $$

where the Fourier coefficients $f_{n} (r)$ can be found from

14 $$ f_{n} (r) = \frac{1}{2\pi }\int\limits_{0}^{2\pi } {f(r,\theta )e^{ - in\theta } d\theta } . $$

The process of finding $f_{n} (r)$ from $f(r,\theta )$ can be thought of as a “forward Fourier series transform” where the continuous $\theta$ variable is transformed to the discrete (although infinite) $n$ variable. The reverse transform is the process of performing the summation over the discrete *n* variable, as given in Eq. (13), to recover the original function as a continuous function of $\theta$. The use of the Discrete Fourier Transform (DFT) via fast algorithms such as the FFT (Cooley and Tukey 1965) to numerically compute the continuous, infinite expressions of (13) and (14) has received much attention in the literature. There is a large body of work on how well the continuous Fourier transform or series can be approximated with the discrete FFT, for example in Epstein (2005).

A transform that is quite useful in 2D polar coordinates is the Hankel transform of order *n*, defined by the integral (Chirikjian and Kyatkin 2000)

15 $$ \hat{f}^{\left( n \right)} \left( \rho \right) = {\mathbb{H}}_{n} \left( {f(r)} \right) = \int\limits_{0}^{\infty } {f(r)J_{n} (\rho r)rdr} , $$

where $J_{n} (z)$ is the *n*th order Bessel function. In 2D coordinates, the overhat symbol $\hat{f}$ is used to denote a Hankel transform. The superscript $\left( n \right)$ is used as a reminder of the order of the Hankel transform—in this case an *n*th order Hankel transform. If $n > - 1/2$, the transform is self-reciprocating and the inversion formula is given by

16 $$ f(r) = {\mathbb{H}}_{n}^{ - 1} \left\{ {\hat{f}^{\left( n \right)} \left( \rho \right)} \right\} = \int\limits_{0}^{\infty } {\hat{f}^{\left( n \right)} (\rho )J_{n} (\rho r)\rho d\rho } . $$

The Laplacian in 2D polar coordinates is given by

17 $$ \nabla^{2} = \frac{{\partial^{2} }}{{\partial r^{2} }} + \frac{1}{r}\frac{\partial }{\partial r} + \frac{1}{{r^{2} }}\frac{{\partial^{2} }}{{\partial \theta^{2} }} $$

For a 2D function in polar coordinates as given by (13), the Laplacian gives

18 $$ \nabla^{2} f\left( {\vec{r}} \right) = \sum\limits_{n = - \infty }^{\infty } {\left( {\frac{{d^{2} f_{n} }}{{dr^{2} }} + \frac{1}{r}\frac{{df_{n} }}{dr} - \frac{{n^{2} f_{n} }}{{r^{2} }}} \right)\;e^{in\theta } } $$

Hence, for a function written in the form of Eq. (13), the required form of the Laplacian is denoted with $\nabla_{n}^{2}$ where this operator is defined by

19 $$ \nabla_{n}^{2} = \frac{{d^{2} }}{{dr^{2} }} + \frac{1}{r}\frac{d}{dr} - \frac{{n^{2} }}{{r^{2} }} . $$

Consider the application of an *n*th order Hankel transform to the 2D Laplacian $\nabla_{n}^{2}$ of any function of *r* only, ${\mathbb{H}}_{n} \left\{ {\nabla_{n}^{2} g(r)} \right\}$:

20 $$ \begin{aligned} {\mathbb{H}}_{n} \left\{ {\nabla_{n}^{2} g(r)} \right\} & = \int\limits_{0}^{\infty } {\left( {\frac{{d^{2} g\left( r \right)}}{{dr^{2} }} + \frac{1}{r}\frac{dg\left( r \right)}{dr} - \frac{{n^{2} g\left( r \right)}}{{r^{2} }}} \right)J_{n} (\rho r)\;rdr} \\ & = - \rho^{2} \int\limits_{0}^{\infty } {g\left( r \right)J_{n} (\rho r)\;rdr} = - \rho^{2} \hat{g}^{\left( n \right)} \left( \rho \right) \\ \end{aligned} $$

where the second line follows from a simple application of integration by parts and the definition of the *n*th order Hankel transform. In other words, the *n*th order Hankel transform of the *n*th order Laplacian of any function $g\left( r \right)$ is the product of $- \rho^{2}$ and the *n*th order Hankel transform of $g\left( r \right)$. As is familiar from Fourier theory, derivatives transform to products under a suitable choice of integral transform. It is noted that the use of the Laplacian $\nabla_{n}^{2}$ necessitates an *n*th order Hankel transform to give the desired simple result in (20).

### Fourier shell identity in 2D

To proceed with the derivation in 2D, the equation for pressure (3) is again considered, and only the forward Fourier series transform is taken. Specifically, $\tilde{p}_{n} \left( {r,k} \right)$ are the Fourier series coefficients of $\tilde{p}\left( {r,\theta ,k} \right)$ via (14) and similarly $A_{n} \left( r \right)$ are the Fourier series coefficients of $A\left( r \right)$, also via Eq. (14). Hence, from (3), the relationship between them is given by

21 $$ \nabla_{n}^{2} \tilde{p}_{n} (r,k) + k^{2} \tilde{p}_{n} (r,k) = - \frac{{ikc_{s} \beta }}{{C_{p} }}\tilde{I}\left( k \right)A_{n} \left( r \right) $$

It is shown in the “Appendix” that the Fourier shell identity stated in 2D is given by

22 $$ J_{n} \left( {kr_{0} } \right)\;r_{0} \frac{d}{dr}\,\tilde{p}_{n} (r_{0} ,k) - \tilde{p}_{n} (r_{0} ,k)r_{0} \frac{d}{dr}J_{n} (kr_{0} ) = - \frac{{ikc_{s} \beta }}{{C_{p} }}\tilde{I}\left( k \right)\hat{A}_{n}^{\left( n \right)} \left( k \right) $$

In Eq. (22), $\hat{A}_{n}^{\left( n \right)} \left( k \right)$ is the *n*th order Hankel transform of $A_{n} \left( r \right)$ evaluated at $\rho = k$ and $r = r_{0}$ is the location where a measurement is made. The tilde refers to a temporal Fourier transform, the overhat refers to a forward Hankel transform and the superscript (*n*) indicates the order of the transform. Equation (22) is the 2D statement of Eq. (4), the Fourier shell identity, where it is noted that the value of $\tilde{p}_{n} (r_{0} ,k)$ and its derivative in the radial direction are required.

### Multivariable Fourier analysis

From the equation for pressure (3), two dimensional polar coordinates are assumed so that position is a function of radius and angle, $\vec{r} = \left( {r,\theta } \right)$. Then, the forward Fourier series transform it taken, followed by an *n*th order Hankel transform. Specifically, $\tilde{p}\left( {r,\theta ,k} \right)$ transforms to $\tilde{p}_{n} \left( {r,k} \right)$ via (14), which in turn transforms to $\hat{\tilde{p}}_{n}^{\left( n \right)} \left( {\rho ,k} \right)$ via (15), an *n*th order Hankel transform of the *n*th term in the Fourier series for $\tilde{p}\left( {r,\theta ,k} \right)$. Symbolically, $\tilde{p}\left( {r,\theta ,k} \right) \to \tilde{p}_{n} \left( {r,k} \right) \to \hat{\tilde{p}}_{n}^{\left( n \right)} \left( {\rho ,k} \right)$. The same applies to the absorber function so that $A\left( {r,\theta } \right) \to A_{n} \left( r \right) \to \hat{A}_{n}^{\left( n \right)} \left( \rho \right)$, and furthermore the Laplacian transforms under the same set of operations as $\nabla^{2} \to \nabla_{n}^{2} \to - \rho^{2}$. The overhat with superscript $\left( n \right)$ denotes an *n*th order Hankel transform, and the subscript of *n* indicates the *n*th term of the Fourier series. Taking transforms and then cleaning up gives

23 $$ \begin{aligned} - \rho^{2} \hat{\tilde{p}}_{n}^{\left( n \right)} \left( {\rho ,k} \right) + k^{2} \hat{\tilde{p}}_{n}^{\left( n \right)} \left( {\rho ,k} \right) = - \frac{{ik\beta c_{s} }}{{C_{p} }}\tilde{I}\left( k \right)\hat{A}_{n}^{\left( n \right)} \left( \rho \right) \hfill \\ \;\;\;\;\;\;\;\;\;\;\;\;\hat{\tilde{p}}_{n}^{\left( n \right)} \left( {\rho ,k} \right) = \frac{{ik\beta c_{s} }}{{C_{p} }}\tilde{I}\left( k \right)\frac{{\hat{A}_{n}^{\left( n \right)} \left( \rho \right)}}{{\rho^{2} - k^{2} }} \hfill \\ \end{aligned} $$

### Fourier shell theorem in 2D polar coordinates

It is shown in Baddour (2011) that the following result holds true

24 $$ \int\limits_{0}^{\infty } {\frac{\phi (\rho )}{{\rho^{2} - k_{{}}^{2} }}} \;J_{n} \left( {\rho r} \right)\rho d\rho = - \pi i\frac{1}{2}H_{n}^{(2)} \left( {kr} \right)\phi \left( k \right) $$

Here, $\phi$ is an analytic function defined on the positive real line that remains bounded as *x* goes to infinity (has no poles), $H_{n}^{(2)} \left( x \right)$ is a Hankel function of order *n.* Given the definition of the Fourier transform that is being currently used, the result in (24) satisfies the Sommerfeld radiation condition, ensuring an outwardly propagating wave.

Returning to Eq. (23), the inverse Hankel transform can be taken the help of the theorem shown in Eq. (24). Assuming that $\hat{A}_{n}^{\left( n \right)} \left( \rho \right)$ has no poles and remains bounded, then

25 $$ \begin{aligned} \tilde{p}_{n} \left( {r,k} \right) & = \frac{{\beta c_{s} ik}}{{C_{p} }}\tilde{I}\left( k \right)\int\limits_{0}^{\infty } {\frac{{\hat{A}_{n}^{\left( n \right)} \left( \rho \right)}}{{\rho^{2} - k^{2} }}J_{n} (\rho r)\rho d\rho } \\ & = \frac{{\beta c_{s} k\pi }}{{2C_{p} }}\tilde{I}\left( k \right)H_{n}^{(2)} \left( {kr} \right)\hat{A}_{n}^{\left( n \right)} \left( k \right) \\ \end{aligned} $$

Equation (25) is the statement of the Fourier shell theorem in 2D. This result if very powerful as it relates the temporal Fourier components of pressure $\tilde{p}_{n} \left( {r,k} \right)$ to the Hankel components of $\hat{A}_{n}^{\left( n \right)} \left( k \right)$. Equation (25) is also easily invertible. Meaning, given measurements made at some detector location $r = r_{0}$, then

26 $$ \hat{A}_{n}^{\left( n \right)} \left( k \right) = \frac{{2C_{p} \tilde{p}_{n} \left( {r_{0} ,k} \right)}}{{\beta c_{s} k\pi \tilde{I}\left( k \right)H_{n}^{(2)} \left( {kr_{0} } \right)}} $$

Hence, the success in being able to reconstruct $A\left( {\vec{r}} \right) = A\left( {r,\theta } \right)$ is partly in the ability to gather sufficient (*k* intensive) information about $\hat{A}_{n}^{\left( n \right)} \left( k \right)$ to enable the inverse Hankel transform to be computed via

27 $$ A_{n} \left( r \right) = \int\limits_{0}^{\infty } {\hat{A}_{n}^{\left( n \right)} (k)J_{n} (kr)kdk} = \frac{{2C_{p} }}{{\beta c_{s} \pi }}\int\limits_{0}^{\infty } {\frac{{\tilde{p}_{n} \left( {r_{0} ,k} \right)}}{{\tilde{I}\left( k \right)H_{n}^{(2)} \left( {kr_{0} } \right)}}J_{n} (kr)dk} $$

Equations (26) and (27) require measurements at a sufficiently wide bandwidth to enable the integral in (27) to be computed or approximated. The second challenge is the computation of a sufficient number of $A_{n} \left( r \right)$ terms (sufficient *n*) to enable $A\left( {\vec{r}} \right) = A\left( {r,\theta } \right)$ to be reconstructed from (or an approximation to)

28 $$ A(r,\theta ) = \sum\limits_{n = - \infty }^{\infty } {A_{n} (r)e^{in\theta } } $$

From (27), a sufficient number of $A_{n} \left( r \right)$ terms is a consequence of a sufficient number of $\tilde{p}_{n} \left( {r_{0} ,k} \right)$ terms, which means that sufficient angular data must be collected to enable $\tilde{p}_{n} \left( {r_{0} ,k} \right)$ to be calculated or approximated from

29 $$ \tilde{p}_{n} (r_{0} ,k) = \frac{1}{2\pi }\int\limits_{0}^{2\pi } {\tilde{p}\left( {r_{0} ,\theta ,k} \right)e^{ - in\theta } d\theta } $$

### Comparison with the Fourier shell identity

Equation (22) is the Fourier shell identity as put forward by Anastasio et al. (2007), where the temporal Fourier component of pressure values $\tilde{p}_{n} (r,k)$ and its derivative are related to the values of a specified Hankel component, $\hat{A}_{n}^{\left( n \right)} \left( k \right)$. The problem with the formulation in (22) is the requirement for measurements of the derivative of $\tilde{p}_{n} (r,k)$, which does not occur in practice. However, it can be shown that the present formulation, as given in Eq. (25) leads to the same result as in (22). Using the expression for $\tilde{p}_{n} (r,k)$ given in (25), it can be substituted into the left hand side of (22) to evaluate

30 $$ \left. {J_{n} \left( {kr_{0} } \right)\;r_{0} \frac{d}{dr}\,\tilde{p}_{n} (r_{0} ,k) - \tilde{p}_{n} (r_{0} ,k)r_{0} \frac{d}{dr}J_{n} (kr_{0} )} \right|_{{\tilde{p}_{n} \left( {r_{0} ,k} \right)\, = \,\frac{{\beta c_{s} k\pi }}{{2C_{p} }}\tilde{I}\left( k \right)H_{n}^{(2)} \left( {kr_{0} } \right)\hat{A}_{n}^{\left( n \right)} \left( k \right)}} $$

which simplifies to

31 $$ \frac{{\beta c_{s} k^{2} \pi r_{0} }}{{2C_{p} }}\tilde{I}\left( k \right)\hat{A}_{n}^{\left( n \right)} \left( k \right)\left[ {J_{n + 1} \left( {kr_{0} } \right)H_{n}^{(2)} \left( {kr_{0} } \right) - J_{n} \left( {kr_{0} } \right)H_{n + 1}^{(2)} \left( {kr_{0} } \right)} \right] $$

There exist well known Wronskian relationships for the Bessel functions (Abramowitz and Stegun 1964; Olver et al. 2010), given by

32 $$ J_{n + 1} \left( {kr} \right)H_{n}^{(2)} \left( {kr} \right) - J_{n} \left( {kr} \right)H_{n + 1}^{(2)} \left( {kr} \right) = - \frac{2i}{\pi kr} $$

Hence, using Eq. (32), Eq. (31) simplifies to

33 $$ - \frac{{ikc_{s} \beta }}{{C_{p} }}\tilde{I}\left( k \right)\hat{A}_{n}^{\left( n \right)} \left( k \right) $$

This is exactly the same expression as on the right hand side of (22), which implies that the results derived in the previous section and those derived by Anastasio et al. (2007) are identical. However, the formulation proposed here, namely Eq. (25) provides a useful alternative since no knowledge or measurement of the derivative of pressure is required.

## Analysis in 3D spherical polar coordinates

### 3D spherical polar coordinates

For a 3D function in spherical polar coordinates,$f\left( {\vec{r}} \right) = f\left( {r,\psi ,\theta } \right)$, where $0 \le \psi \le \pi$ represents the colatitude which ranges from 0 at the North Pole, to π/2 at the Equator, to π at the South Pole and $0 \le \theta \le 2\pi$ represents the longitude (azimuth angle). The angular dependence can be expanded into a spherical harmonic series (similar to a Fourier series in 2D polar coordinates) so that the function can be written as

34 $$ f\left( {r,\psi ,\theta } \right) = \sum\limits_{l = 0}^{\infty } {} \sum\limits_{m = - l}^{l} {f_{l}^{m} \left( r \right)Y_{l}^{m} (\psi ,\theta )} $$

where the spherical harmonic coefficients in the series are given by

35 $$ f_{l}^{m} \left( r \right) = \int\limits_{0}^{2\pi } {} \int\limits_{0}^{\pi } {f\left( {r,\psi ,\theta } \right)\overline{{Y_{l}^{m} \left( {\psi ,\theta } \right)}} \sin \psi \;d\psi \;d\theta } . $$

The $Y_{l}^{m} (\psi ,\theta )$ are spherical harmonics, solutions to the angular portion of Laplace’s equation in spherical polar coordinates, and can be shown to be orthogonal. These spherical harmonics are given by (Chirikjian and Kyatkin 2000)

36 $$ Y_{l}^{m} (\psi ,\theta ) = \sqrt {\frac{(2l + 1)(l - m)!}{4\pi (l + m)!}} P_{l}^{m} \left( {\cos \psi } \right)e^{im\theta } $$

where $Y_{l}^{m}$ is a called a spherical harmonic function of degree $l$ and order *m*, and $P_{l}^{m}$ is an associated Legendre function. The coefficients $f_{l}^{m} \left( r \right)$ are sometimes referred to as the *spherical Fourier transform* of $f\left( {r,\psi ,\theta } \right)$ (Telenkov et al. 2009). The process of finding $f_{l}^{m} \left( r \right)$ from $f\left( {r,\psi ,\theta } \right)$ can be thought of as a “forward spherical Fourier series transform” where the continuous $\left( {\psi ,\theta } \right)$ variables are transformed to the discrete (although infinite) $m,l$ variables. The reverse transform is the process of performing the summation over the discrete $m,l$ variables, as given in (34), to recover the original continuous function.

A transform that is useful in spherical polar coordinates is the spherical Hankel transform and its inverse. These are defined as (Bracewell 1999; Piessens 2000)

37 $$ \hat{f}^{\left( n \right)} \left( \rho \right) = {\mathbb{S}}_{n} \left\{ {f\left( r \right)} \right\} = \int\limits_{0}^{\infty } {f\left( r \right)j_{n} \left( {\rho r} \right)r^{2} dr} \quad f\left( r \right) = \frac{2}{\pi }\int\limits_{0}^{\infty } {\hat{f}^{\left( n \right)} \left( \rho \right)j_{n} \left( {\rho r} \right)\rho^{2} d\rho } . $$

${\mathbb{S}}_{n}$ is used to specifically denote the spherical Hankel transform of order *n*. The spherical Hankel transform is particularly useful for problems involving spherical symmetry.

The Laplacian in 3D spherical polar coordinates is given by

38 $$ \nabla^{2} = \frac{{\partial^{2} }}{{\partial r^{2} }} + \frac{2}{r}\frac{\partial }{\partial r} + \frac{1}{{r^{2} \sin \psi }}\frac{\partial }{\partial \psi }\left( {\sin \psi \frac{\partial }{\partial \psi }} \right) + \frac{1}{{r^{2} \sin^{2} \psi }}\frac{{\partial^{2} }}{{\partial \theta^{2} }} $$

For a 3D function in spherical coordinates as given by (34), the Laplacian simplifies to

39 $$ \begin{aligned} \nabla^{2} f\left( {\vec{r}} \right) & = \left( {\frac{{\partial^{2} }}{{\partial r^{2} }} + \frac{2}{r}\frac{\partial }{\partial r} + \frac{1}{{r^{2} \sin \psi }}\frac{\partial }{\partial \psi }\left( {\sin \psi \frac{\partial }{\partial \psi }} \right) + \frac{1}{{r^{2} \sin^{2} \psi }}\frac{{\partial^{2} }}{{\partial \theta^{2} }}} \right)\sum\limits_{l = 0}^{\infty } {} \sum\limits_{m = - l}^{l} {f_{l}^{m} \left( r \right)Y_{l}^{m} (\psi ,\theta )} \\ & = \sum\limits_{l = 0}^{\infty } {\sum\limits_{m = - l}^{l} {\left( {\frac{{d^{2} }}{{dr^{2} }} + \frac{2}{r}\frac{d}{dr} - \frac{{l\left( {l + 1} \right)}}{{r^{2} }}} \right)f_{l}^{m} \left( r \right)Y_{l}^{m} (\psi ,\theta )} } \\ \end{aligned} $$

Hence, from (39) it can be seen that for a function written in the form of (34), the required form of the 3D Laplacian is denoted with $\nabla_{l}^{2}$ where this operator is defined by

40 $$ \nabla_{l}^{2} = \frac{{d^{2} }}{{dr^{2} }} + \frac{2}{r}\frac{d}{dr} - \frac{{l\left( {l + 1} \right)}}{{r^{2} }}. $$

Consider the application of an *n*th order spherical Hankel transform to the 3D Laplacian $\nabla_{n}^{2}$ of any function of *r* only, ${\mathbb{S}}_{n} \left\{ {\nabla_{n}^{2} g(r)} \right\}$:

41 $$ {\mathbb{S}}_{n} \left\{ {\nabla_{n}^{2} g(r)} \right\} = \int\limits_{0}^{\infty } {\left( {\frac{{d^{2} g\left( r \right)}}{{dr^{2} }} + \frac{2}{r}\frac{dg\left( r \right)}{dr} - \frac{n(n + 1)g\left( r \right)}{{r^{2} }}} \right)j_{n} (\rho r)\;r^{2} dr} $$

A simple application of integration by parts along with the definition of an *n*th order spherical Bessel function gives

42 $$ \begin{aligned} {\mathbb{S}}_{n} \left\{ {\nabla_{n}^{2} g(r)} \right\} & = \int\limits_{0}^{\infty } {\nabla_{n}^{2} g\left( r \right)j_{n} (\rho r)\;r^{2} dr} = - \rho^{2} \int\limits_{0}^{\infty } {g\left( r \right)j_{n} (\rho r)\;r^{2} dr} \\ & = - \rho^{2} {\mathbb{S}}_{n} \left\{ {g(r)} \right\} = - \rho^{2} \hat{g}^{\left( n \right)} \left( \rho \right) \\ \end{aligned} $$

In other words, the *n*th order spherical Hankel transform of the *n*th order 3D Laplacian of $g\left( r \right)$ is the product of $- \rho^{2}$ and the *n*th order spherical Hankel transform of *g*. As seen before, derivatives transform to products under the appropriate choice of transform.

### Fourier shell theorem in 3D spherical polar coordinates

The analysis returns to the equation for pressure, (3) and proceeds by taking only the forward spherical harmonic transform. Specifically, $\tilde{p}\left( {r,\psi ,\theta ,k} \right)$ transforms to $\tilde{p}_{l}^{m} \left( {r,k} \right)$ via (35), the Laplacian transform to $\nabla_{l}^{2}$ and finally $A\left( {r,\psi ,\theta } \right)$ transform to $A_{l}^{m\;} \left( r \right)$, also via Eq. (35). This gives

43 $$ \nabla_{l}^{2} \tilde{p}_{l}^{m\;} \left( {r,k} \right) + k^{2} \tilde{p}_{l}^{m\;} \left( {r,k} \right) = - \frac{{ik\beta c_{s} }}{{C_{p} }}\tilde{I}\left( k \right)A_{l}^{m\;} \left( r \right) $$

It is shown in the “Appendix” that the Fourier shell identity stated in 3D is given by

44 $$ j_{l} \left( {kr_{0} } \right)r_{0}^{2} \frac{d}{dr}\tilde{p}_{l}^{m\;} \left( {r_{0} ,k} \right)\; - \tilde{p}_{l}^{m\;} \left( {r_{0} ,k} \right)r_{0}^{2} \frac{d}{dr}j_{l} \left( {kr_{0} } \right) = - \frac{{ikc_{s} \beta }}{{C_{p} }}\tilde{I}\left( k \right)\hat{A}_{l}^{m\left( l \right)} \left( k \right) $$

Equation (44) is 3D statement of Eq. (4), the Fourier shell identity. In (44), the tilde refers to a temporal Fourier transform, the overhat refers to a forward spherical Hankel transform, and the $\left( l \right)$ superscript refers to order of the spherical Hankel transform.

### Multivariable Fourier analysis

Taking the temporal Fourier transform of (1) and assuming three dimensional spherical polar coordinates so that position is a function of radius and angle, $\vec{r} = \left( {r,\psi ,\theta } \right)$, it follows that

45 $$ \nabla^{2} \tilde{p}\left( {r,\psi ,\theta ,k} \right) + k^{2} \tilde{p}\left( {r,\psi ,\theta ,k} \right) = - \frac{{ik\beta c_{s} }}{{C_{p} }}\tilde{I}\left( k \right)A\left( {r,\psi ,\theta } \right) $$

where $k = \omega /c_{s}$ is the wave number. Now take the forward spherical harmonic transform, followed by an *n*th order spherical Hankel transform of Eq. (45). Specifically, $\tilde{p}\left( {r,\psi ,\theta ,k} \right)$ transforms to $\tilde{p}_{l}^{m} \left( {r,k} \right)$ via (35), which in turn transforms to $\hat{\tilde{p}}_{l}^{m\;\left( l \right)} \left( {\rho ,k} \right)$ via (37), an *l*th order spherical Hankel transform of the $_{l}^{m}$ th term in the spherical harmonic series for $\tilde{p}\left( {r,\psi ,\theta ,k} \right)$. Symbolically, $\tilde{p}\left( {r,\psi ,\theta ,k} \right) \to \tilde{p}_{l}^{m} \left( {r,k} \right) \to \hat{\tilde{p}}_{l}^{m\;\left( l \right)} \left( {\rho ,k} \right)$. The same applies to $A\left( {r,\psi ,\theta } \right) \to A_{l}^{m} \left( r \right) \to \hat{A}_{l}^{m\;\left( l \right)} \left( \rho \right)$ and the Laplacian transforms under the same set of operations as $\nabla^{2} \to \nabla_{l}^{2} \to - \rho^{2}$. The overhat with superscript $\left( l \right)$ denotes an *l*th order spherical Hankel transform, and the $_{l}^{m}$ indicates the $_{l}^{m}$ th term of the spherical hamornic series. Taking transforms and then cleaning up Eq. (45) gives

46 $$ \begin{aligned} - \rho^{2} \hat{\tilde{p}}_{l}^{m\;\left( l \right)} \left( {\rho ,k} \right) + k^{2} \hat{\tilde{p}}_{l}^{m\;\left( l \right)} \left( {\rho ,k} \right) = - \frac{{ik\beta c_{s} }}{{C_{p} }}\tilde{I}\left( k \right)\hat{A}_{l}^{m\;\left( l \right)} \left( \rho \right) \hfill \\ \;\;\;\;\;\;\;\;\;\;\;\;\hat{\tilde{p}}_{l}^{m\;\left( l \right)} \left( {\rho ,k} \right) = \frac{{ik\beta c_{s} }}{{C_{p} }}\tilde{I}\left( k \right)\frac{{\hat{A}_{l}^{m\;\left( l \right)} \left( \rho \right)}}{{\rho^{2} - k^{2} }} \hfill \\ \end{aligned} $$

### Fourier shell theorem in 3D polar coordinates

It is shown in Baddour (2011) that the following result holds true

47 $$ \int\limits_{0}^{\infty } {\frac{\phi (\rho )}{{\rho^{2} - k^{2} }}} j_{n} \left( {\rho r} \right)\rho^{2} d\rho = - \pi i\frac{k}{2}h_{n}^{(2)} \left( {kr} \right)\phi \left( k \right) $$

Here, $\phi$ is an analytic function defined on the positive real line that remains bounded as *x* goes to infinity (has no poles), $h_{n}^{(2)} \left( x \right)$ is a spherical Hankel function of order *n.* Given the definition of the Fourier transform that is being currently used, the presented result satisfies the Sommerfeld radiation condition, ensuring an outwardly propagating wave.

Returning to Eq. (46), the inverse spherical Hankel transform can be taken with the help of the theorem in Eq. (47). Assuming that $\hat{A}_{l}^{m\;\left( l \right)} \left( \rho \right)$ has no poles and remains bounded, then

48 $$ \begin{aligned} \tilde{p}_{l}^{m} \left( {r,k} \right) & = \frac{2}{\pi }\int\limits_{0}^{\infty } {\hat{\tilde{p}}_{l}^{m\;\left( l \right)} \left( {\rho ,k} \right)j_{l} (\rho r)\rho^{2} d\rho } = \frac{{2ik\beta c_{s} }}{{\pi C_{p} }}\tilde{I}\left( k \right)\int\limits_{0}^{\infty } {\frac{{\hat{A}_{l}^{m\;\left( l \right)} \left( \rho \right)}}{{\rho^{2} - k^{2} }}j_{l} (\rho r)\rho^{2} d\rho } \\ & = \frac{{k^{2} \beta c_{s} }}{{C_{p} }}\tilde{I}\left( k \right)h_{l}^{(2)} \left( {kr} \right)\hat{A}_{l}^{m\;\left( l \right)} \left( k \right) \\ \end{aligned} $$

Equation (48) is the statement of the Fourier shell theorem in 3D. This result if very powerful as it relates the temporal Fourier components of pressure $\tilde{p}_{l}^{m\;\left( l \right)} \left( {r,k} \right)$ to the Hankel components of $\hat{A}_{l}^{m\;\left( l \right)} \left( k \right)$. Equation (48) is also easily invertible. Meaning, given measurements made at some detector location $r = r_{0}$, then

49 $$ \hat{A}_{l}^{m\;\left( l \right)} \left( k \right) = \frac{{C_{p} \tilde{p}_{l}^{m} \left( {r_{0} ,k} \right)}}{{k^{2} \beta c_{s} \tilde{I}\left( k \right)h_{l}^{(2)} \left( {kr_{0} } \right)}} $$

Hence, the success in being able to reconstruct $A\left( {\vec{r}} \right) = A\left( {r,\psi ,\theta } \right)$ is partly in the ability to gather sufficient (*k* intensive) information about $\hat{A}_{l}^{m\;\left( l \right)} \left( k \right)$ to enable the inverse spherical Hankel transform to be computed via

50 $$ A_{l}^{m\;} \left( r \right) = \frac{2}{\pi }\int\limits_{0}^{\infty } {\hat{A}_{l}^{m\;\left( l \right)} \left( k \right)j_{l} (kr)kdk} = \frac{2}{\pi }\int\limits_{0}^{\infty } {\frac{{C_{p} \tilde{p}_{l}^{m} \left( {r_{0} ,k} \right)}}{{k^{2} \beta c_{s} \tilde{I}\left( k \right)h_{l}^{(2)} \left( {kr_{0} } \right)}}j_{l} (kr)kdk} $$

Equations (49) and (50) require measurements at a sufficiently wide bandwidth to enable the integral in (50) to be computed or approximated. The second challenge is the computation of a sufficient number of $A_{l}^{m\;} \left( r \right)$ terms (sufficient *m, l*) to enable $A\left( {\vec{r}} \right) = A\left( {r,\psi ,\theta } \right)$ to be reconstructed from

51 $$ A\left( {r,\psi ,\theta } \right) = \sum\limits_{l = 0}^{\infty } {} \sum\limits_{m = - l}^{l} {A_{l}^{m} \left( r \right)Y_{l}^{m} (\psi ,\theta )} $$

From (49), a sufficient number of $A_{l}^{m\;} \left( r \right)$ terms is a consequence of a sufficient number of $\tilde{p}_{l}^{m} \left( {r_{0} ,k} \right)$ terms, which means that sufficient angular data must be collected to enable $\tilde{p}_{l}^{m} \left( {r_{0} ,k} \right)$ to be calculated or approximated from

52 $$ \tilde{p}_{l}^{m\;} \left( {r_{0} ,k} \right) = \int\limits_{0}^{2\pi } {} \int\limits_{0}^{\pi } {\tilde{p}\left( {r_{0} ,\psi ,\theta ,k} \right)\overline{{Y_{l}^{m} \left( {\psi ,\theta } \right)}} \sin \psi \;d\psi \;d\theta } $$

### Comparison with the Fourier shell identity

Equation (44) is the 3D version of the “Fourier shell identity” as put forward by Anastasio et al. (2007), where the temporal Fourier component of pressure values $\tilde{p}_{l}^{m} \left( {r,k} \right)$ and its derivative are related to the values of a specified Hankel component, $\hat{A}_{l}^{m\;\left( l \right)} \left( k \right)$. The problem with the formulation in (44) is the requirement for measurements of the derivative of $\tilde{p}_{n} (r,k)$, which does not occur in practice. However, it can be shown that the present formulation, as given in Eq. (48) leads to the same result as in (44). Using the expression for $\tilde{p}_{l}^{m} \left( {r,k} \right)$ given in (48), it can be substituted into the left hand side of (44) to evaluate

53 $$ \left. {j_{l} \left( {kr_{0} } \right)r_{0}^{2} \frac{d}{dr}\tilde{p}_{l}^{m\;} \left( {r_{0} ,k} \right)\; - \tilde{p}_{l}^{m\;} \left( {r_{0} ,k} \right)r_{0}^{2} \frac{d}{dr}j_{l} \left( {kr_{0} } \right)} \right|_{{\tilde{p}_{l}^{m\;} \left( {r_{0} ,k} \right)\,=\,\frac{{k^{2} \beta c_{s} }}{{C_{p} }}\tilde{I}\left( k \right)h_{l}^{(2)} \left( {kr_{0} } \right)\hat{A}_{l}^{m\;\left( l \right)} \left( k \right)}} $$

which simplifies to

54 $$ \begin{aligned} \frac{{k^{2} \beta c_{s} \tilde{I}\left( k \right)\hat{A}_{l}^{m\;\left( l \right)} \left( k \right)}}{{C_{p} }}r_{0}^{2} \left\{ {j_{l} \left( {kr_{0} } \right)\frac{d}{dr}h_{l}^{(2)} \left( {kr_{0} } \right) - h_{l}^{(2)} \left( {kr_{0} } \right)\frac{d}{dr}j_{l} \left( {kr_{0} } \right)} \right\} \hfill \\ = \frac{{k^{3} \beta c_{s} \tilde{I}\left( k \right)\hat{A}_{l}^{m\;\left( l \right)} \left( k \right)}}{{C_{p} }}r_{0}^{2} \left\{ {h_{l}^{(2)} \left( {kr_{0} } \right)j_{l + 1} \left( {kr_{0} } \right) - j_{l} \left( {kr_{0} } \right)h_{l + 1}^{(2)} \left( {kr_{0} } \right)} \right\} \hfill \\ \end{aligned} $$

Making us of the well-known Wronskian relationship for spherical Bessel functions (Abramowitz and Stegun 1964; Olver et al. 2010) given by

55 $$ h_{l}^{(2)} \left( {kr} \right)j_{l + 1} \left( {kr} \right) - j_{l} \left( {kr} \right)h_{l + 1}^{(2)} \left( {kr} \right) = - \frac{i}{{k^{2} r^{2} }} $$

then Eq. (54) simplifies to

56 $$ - \frac{{ik\beta c_{s} \tilde{I}\left( k \right)\hat{A}_{l}^{m\;\left( l \right)} \left( k \right)}}{{C_{p} }} $$

Equation (56) is exactly the same expression as on the right hand side of (44), which implies that the results derived in the previous section and those derived by Anastasio et al. (2007) are identical. However, the formulation of the Fourier shell theorem proposed here, namely Eq. (48) provides a useful alternative since no knowledge or measurement of the derivative of pressure is required.

## Summary and conclusions

In this paper, re-derived and derivative-free formulations of the Fourier shell identity were presented in 1D, 2D polar and 3D spherical polar coordinates. These were shown to be equivalent to the previously derived Fourier shell identity (Anastasio et al. 2007), however knowledge of the derivative of pressure is not required, hence lending the formulas directly applicable to Fourier-based absorber reconstruction schemes. The formulas are restated here as a summary.

In 1D, the Fourier shell identity is given by

57 $$ \tilde{p}\left( {z,k} \right) = \frac{{\beta c_{s} }}{{2C_{p} }}\tilde{I}\left( k \right)\left\{ {\begin{array}{*{20}l} {\hat{A}\left( { - k} \right)e^{ - ikz} } \hfill & {z > 0} \hfill \\ {\hat{A}\left( k \right)e^{ikz} } \hfill & {z < 0} \hfill \\ \end{array} } \right. $$

In Eq. (57), the $\sim$ refers to a temporal Fourier transform and the overhat refers to a 1D spatial Fourier transform.

In 2D polar coordinates, the Fourier shell identity is given by

58 $$ \tilde{p}_{n} \left( {r,k} \right) = \frac{{\beta c_{s} k\pi }}{{2C_{p} }}\tilde{I}\left( k \right)H_{n}^{(2)} \left( {kr} \right)\hat{A}_{n}^{\left( n \right)} \left( k \right) $$

In Eq. (58), the *n* subscript refers to the *n*th Fourier coefficient in the Fourier series. The overhat refers to a forward Hankel transform, the superscript (*n*) refers to the order of the Hankel transform and $H_{n}^{(2)} \left( x \right)$ is a Hankel function of order *n*.

In 3D spherical polar coordinates, the Fourier shell identity is given by

59 $$ \tilde{p}_{l}^{m} \left( {r,k} \right)\; = \frac{{k^{2} \beta c_{s} }}{{C_{p} }}\tilde{I}\left( k \right)h_{l}^{(2)} \left( {kr} \right)\hat{A}_{l}^{m\;\left( l \right)} \left( k \right) $$

In Eq. (59), the $_{l}^{m}$ subscript/superscript refer to the $_{l}^{m}$ term in the spherical harmonic series expansion, the overhat refers to a forward spherical Hankel transform, the $\left( l \right)$ superscript refers to order of the spherical Hankel transform and $h_{n}^{(2)} \left( x \right)$ is a spherical Hankel function of order *n*.

## Appendix: Proofs

### The Fourier shell theorem in 1D

By following the same procedure used in Anastasio et al. (2007), it can be shown that the Fourier shell identity result presented in “Fourier shell identity in 1D” section is consistent with the result as derived by Anastasio et al. (2007). Anastasio et al. wrote their proof in very general terms and here we make it concrete for the 1D Cartesian coordinate case.

Starting with the equation for pressure in the frequency domain:

60 $$ \frac{{d^{2} }}{{dz^{2} }}\tilde{p}\left( {z,k} \right) + k^{2} \tilde{p}\left( {z,k} \right) = - \frac{{ik\beta c_{s} }}{{C_{p} }}\tilde{I}\left( k \right)A\left( z \right) $$

Furthermore, the complex exponentials $e^{ \pm ikz}$ both satisfy the Helmholtz equation:

61 $$ \frac{{d^{2} }}{{dz^{2} }}e^{ \pm ikz} + k^{2} e^{ \pm ikz} = 0 $$

Multiplying Eq. (60) by $e^{ - ikz}$ and Eq. (61) by $\tilde{p}(z,k)$ and subtracting gives

62 $$ e^{ - ikz} \frac{{d^{2} }}{{dz^{2} }}\tilde{p}\left( {z,k} \right) - \tilde{p}(z,k)\frac{{d^{2} }}{{dz^{2} }}e^{ - ikz} = - \frac{{ik\beta c_{s} }}{{C_{p} }}\tilde{I}\left( k \right)A\left( z \right)e^{ - ikz} $$

Integrating both sides from $- z_{0}$ to $z_{0}$ and using integration by parts gives

63 $$ \int\limits_{{ - z_{0} }}^{{z_{0} }} {} e^{ - ikz} \frac{{d^{2} }}{{dz^{2} }}\tilde{p}\left( {z,k} \right) - \tilde{p}(z,k)\frac{{d^{2} }}{{dz^{2} }}e^{ - ikz} dz = - \frac{{ik\beta c_{s} }}{{C_{p} }}\tilde{I}\left( k \right)\int\limits_{{ - z_{0} }}^{{z_{0} }} {} A\left( z \right)e^{ - ikz} dz $$

which becomes

64 $$ e^{ - ikz} \frac{d}{dz}\tilde{p}\left( {z,k} \right) - \tilde{p}(z,k)\left. {\frac{d}{dz}e^{ - ikz} } \right|_{{ - z_{0} }}^{{z_{0} }} = - \frac{{ik\beta c_{s} }}{{C_{p} }}\tilde{I}\left( k \right)\int\limits_{{ - z_{0} }}^{{z_{0} }} {} A\left( z \right)e^{ - ikz} dz $$

or

65 $$ \begin{aligned} e^{{ - ikz_{0} }} \frac{d}{dz}\tilde{p}\left( {z_{0} ,k} \right) + ik\tilde{p}(z_{0} ,k)e^{{ - ikz_{0} }} - e^{{ikz_{0} }} \frac{d}{dz}\tilde{p}\left( { - z_{0} ,k} \right) - ik\tilde{p}( - z_{0} ,k)e^{{ikz_{0} }} \hfill \\ = - \frac{{ik\beta c_{s} }}{{C_{p} }}\tilde{I}\left( k \right)\int\limits_{{ - z_{0} }}^{{z_{0} }} {} A\left( z \right)e^{ - ikz} dz \hfill \\ \end{aligned} $$

It was assumed in Anastasio et al. (2007) that the measurement surface $\varOmega_{0}$, which is here $\pm z_{0}$, completely encloses the source function. This means that $A\left( z \right)$ has compact support and the integration limits on the right hand side of (65) can be taken to infinite without loss of generality. This implies that

66 $$ \int\limits_{{ - z_{0} }}^{{z_{0} }} {} A\left( z \right)e^{ - ikz} dz = \int\limits_{ - \infty }^{\infty } {} A\left( z \right)e^{ - ikz} dz = \hat{A}\left( k \right)\;\; $$

where the overhat indicates a spatial Fourier transform and the transform $\hat{A}\left( {\omega_{z} } \right)$ is evaluated at $\omega_{z} = k$. Under this assumption of the measurement surface enclosing the source, then

67 $$ e^{{ - ikz_{0} }} \frac{d}{dz}\tilde{p}\left( {z_{0} ,k} \right) + ik\tilde{p}(z_{0} ,k)e^{{ - ikz_{0} }} - e^{{ikz_{0} }} \frac{d}{dz}\tilde{p}\left( { - z_{0} ,k} \right) - ik\tilde{p}( - z_{0} ,k)e^{{ikz_{0} }} = - \frac{{ik\beta c_{s} }}{{C_{p} }}\tilde{I}\left( k \right)\hat{A}\left( k \right) $$

which can be rearranged as

68 $$ \hat{A}\left( k \right) = \frac{{iC_{p} }}{{k\beta c_{s} \tilde{I}\left( k \right)}}\left[ {e^{{ - ikz_{0} }} \left( {\frac{d}{dz}\tilde{p}\left( {z_{0} ,k} \right) + ik\tilde{p}(z_{0} ,k)} \right) - e^{{ikz_{0} }} \left( {\frac{d}{dz}\tilde{p}\left( { - z_{0} ,k} \right) + ik\tilde{p}( - z_{0} ,k)} \right)} \right] $$

Equation (68) is 1D equivalent of Eq. (4), the Fourier shell identity. Equation (68) at first appears more complicated than Anastasio’s formulation. The apparent difficulty is that in it is necessary to enclose the source function. To enclose the source function in 1D, we need to set up a “detecting surface” at *both*$z = \pm z_{0}$ because a detector only at $z = z_{0}$ will not enclose the source.

### The Fourier shell identity in 2D

By following the same procedure used in Anastasio et al. (2007), it can be shown that the Fourier shell identity result given in “Fourier shell identity in 2D” section is consistent with the result as derived by Anastasio et al. (2007). Anastasio et al. wrote their proof in very general terms and here we make it concrete for the 2D polar coordinates.

Starting with the equation for pressure, (3) and taking only the forward Fourier series transform gives

69 $$ \nabla_{n}^{2} \tilde{p}_{n} (r,k) + k^{2} \tilde{p}_{n} (r,k) = - \frac{{ikc_{s} \beta }}{{C_{p} }}\tilde{I}\left( k \right)A_{n} \left( r \right) $$

Furthermore, from the definition of the Bessel functions themselves via Bessel’s equation, the Bessel functions satisfy the homogeneous Helmholtz equation in the forward Fourier series space, such that

70 $$ \left( {\frac{{d^{2} }}{{dr^{2} }}J_{n} (kr) + \frac{1}{r}\frac{d}{dr}J_{n} (kr) - \frac{{n^{2} }}{{r^{2} }}J_{n} (kr)} \right) + k^{2} J_{n} (kr) = \nabla_{n}^{2} J_{n} \left( {kr} \right) + k^{2} J_{n} (kr) = 0 $$

Multiplying Eq. (69) by $J_{n} \left( {kr} \right)$ and Eq. (70) by $\tilde{p}_{n} (r,k)$ and subtracting gives

71 $$ \begin{aligned} J_{n} \left( {kr} \right)\left( {\frac{{d^{2} }}{{dr^{2} }}\tilde{p}_{n} (r,k) + \frac{1}{r}\frac{d}{dr}\tilde{p}_{n} (r,k)} \right) - \tilde{p}_{n} (r,k)\left( {\frac{{d^{2} }}{{dr^{2} }}J_{n} (kr) + \frac{1}{r}\frac{d}{dr}J_{n} (kr)} \right) \hfill \\ = - \frac{{ikc_{s} \beta }}{{C_{p} }}\tilde{I}\left( k \right)A_{n} \left( r \right)J_{n} \left( {kr} \right) \hfill \\ \end{aligned} $$

Multiplying both sides by *r* and using the fact that

72 $$ \frac{d}{dr}\left( {r\frac{d}{dr}} \right) = r\frac{{d^{2} }}{{dr^{2} }} + \frac{d}{dr} $$

gives

73 $$ J_{n} \left( {kr} \right)\frac{d}{dr}\left( {r\frac{d}{dr}\tilde{p}_{n} (r,k)} \right)\; - \tilde{p}_{n} (r,k)\frac{d}{dr}\left( {r\frac{d}{dr}J_{n} (kr)} \right) = - \frac{{ikc_{s} \beta }}{{C_{p} }}\tilde{I}\left( k \right)A_{n} \left( r \right)J_{n} \left( {kr} \right)r $$

Integrating both sides from 0 to some fixed $r_{0}$ and using integration by parts gives

74 $$ J_{n} \left( {kr_{0} } \right)\;r_{0} \frac{d}{dr}\tilde{p}_{n} (r_{0} ,k) - \tilde{p}_{n} (r_{0} ,k)r_{0} \frac{d}{dr}J_{n} (kr_{0} ) = - \frac{ikc\beta }{{C_{p} }}\tilde{I}\left( k \right)\int\limits_{0}^{{r_{0} }} {} A_{n} \left( r \right)J_{n} \left( {kr} \right)rdr $$

It was assumed in Anastasio et al. (2007) that the measurement surface $\varOmega_{0}$, which is here $r = r_{0}$, completely encloses the source function. This means that $A\left( {r,\theta } \right)$ has compact support and the integration limits on the right hand side of (65) can be taken to infinite without loss of generality. This implies that

75 $$ \int\limits_{0}^{{r_{0} }} {} A_{n} \left( r \right)J_{n} \left( {kr} \right)rdr = \int\limits_{0}^{\infty } {} A_{n} \left( r \right)J_{n} \left( {kr} \right)rdr = \hat{A}_{n}^{\left( n \right)} \left( k \right)\;\;\;\;\;\; $$

where the overhat indicates a Hankel transform and the Hankel transform $\hat{A}^{\left( n \right)} \left( \rho \right)$ is evaluated at $\rho = k$. Hence, Eq. (74) becomes

76 $$ J_{n} \left( {kr_{0} } \right)\;r_{0} \frac{d}{dr}\tilde{p}_{n} (r_{0} ,k) - \tilde{p}_{n} (r_{0} ,k)r_{0} \frac{d}{dr}J_{n} (kr_{0} ) = - \frac{{ikc_{s} \beta }}{{C_{p} }}\tilde{I}\left( k \right)\hat{A}_{n}^{\left( n \right)} \left( k \right) . $$

### The Fourier shell identity in 3D

By following the same procedure used in Anastasio et al. (2007), it can be shown that the Fourier shell identity result given in “Fourier shell theorem in 3D spherical polar coordinates” section is consistent with the result as derived by Anastasio et al. (2007). Anastasio et al. wrote their proof in very general terms and here we make it concrete for the 3D spherical polar coordinates.

Starting with the equation for pressure (3) and taking only the forward spherical harmonic transform gives

77 $$ \nabla_{l}^{2} \tilde{p}_{l}^{m\;} \left( {r,k} \right) + k^{2} \tilde{p}_{l}^{m\;} \left( {r,k} \right) = - \frac{{ik\beta c_{s} }}{{C_{p} }}\tilde{I}\left( k \right)A_{l}^{m\;} \left( r \right) $$

Furthermore, from the definition of the spherical Bessel functions themselves via Bessel’s equation, the spherical Bessel functions satisfy the homogeneous Helmholtz equation in the forward spherical harmonic series space, such that

78 $$ \left( {\frac{{d^{2} }}{{dr^{2} }} + \frac{2}{r}\frac{d}{dr} - \frac{{l\left( {l + 1} \right)}}{{r^{2} }}} \right)j_{l} \left( {kr} \right) + k^{2} j_{l} (kr) = \nabla_{l}^{2} j_{l} \left( {kr} \right) + k^{2} j_{l} (kr) = 0 $$

Multiplying Eq. (77) by $j_{l} \left( {kr} \right)$ and Eq. (78) by $\tilde{p}_{n} (r,k)$ and subtracting gives

79 $$ \begin{aligned} j_{l} \left( {kr} \right)\left( {\frac{{d^{2} }}{{dr^{2} }}\tilde{p}_{l}^{m\;} \left( {r,k} \right) + \frac{2}{r}\frac{d}{dr}\tilde{p}_{l}^{m\;} \left( {r,k} \right)} \right) - \tilde{p}_{l}^{m\;} \left( {r,k} \right)\left( {\frac{{d^{2} }}{{dr^{2} }}j_{l} \left( {kr} \right) + \frac{2}{r}\frac{d}{dr}j_{l} \left( {kr} \right)} \right) \hfill \\ \;\;\;\;\;\;\;\;\;\;\;\;\;\;\;\;\;\;\;\;\;\;\;\;\;\;\;\;\;\;\;\;\;\;\;\;\;\;\;\;\;\;\;\;\;\;\;\;\;\;\;\;\;\;\;\;\;\;\;\;\;\;\;\;\; = - \frac{{ikc_{s} \beta }}{{C_{p} }}\tilde{I}\left( k \right)A_{l}^{m} \left( r \right)j_{l} \left( {kr} \right) \hfill \\ \end{aligned} $$

Multiplying both sides by $r^{2}$ and using the fact that

80 $$ \frac{d}{dr}\left( {r^{2} \frac{d}{dr}} \right) = r^{2} \frac{{d^{2} }}{{dr^{2} }} + 2r\frac{d}{dr} $$

gives

81 $$ j_{l} \left( {kr} \right)\frac{d}{dr}\left( {r^{2} \frac{d}{dr}\tilde{p}_{l}^{m\;} \left( {r,k} \right)} \right)\; - \tilde{p}_{l}^{m\;} \left( {r,k} \right)\frac{d}{dr}\left( {r^{2} \frac{d}{dr}j_{l} \left( {kr} \right)} \right) = - \frac{{ikc_{s} \beta }}{{C_{p} }}\tilde{I}\left( k \right)A_{l}^{m} \left( r \right)j_{l} \left( {kr} \right)r^{2} $$

Integrating both sides from 0 to some fixed $r_{0}$ and using integration by parts gives

82 $$ j_{l} \left( {kr_{0} } \right)r_{0}^{2} \frac{d}{dr}\tilde{p}_{l}^{m\;} \left( {r_{0} ,k} \right)\; - \tilde{p}_{l}^{m\;} \left( {r_{0} ,k} \right)r_{0}^{2} \frac{d}{dr}j_{l} \left( {kr_{0} } \right) = - \frac{{ikc_{s} \beta }}{{C_{p} }}\tilde{I}\left( k \right)\int\limits_{0}^{{r_{0} }} {} A_{l}^{m} \left( r \right)j_{l} \left( {kr} \right)r^{2} dr $$

It was assumed in Anastasio et al. (2007) that the measurement surface $\varOmega_{0}$, which is here $r = r_{0}$, completely encloses the source function. This means that $A\left( {r,\psi ,\theta } \right)$ has compact support and the integration limits on the right hand side of (82) can be taken to infinite without loss of generality. It then follows that

83 $$ \int\limits_{0}^{{r_{0} }} {} A_{l}^{m} \left( r \right)j_{l} \left( {kr} \right)r^{2} dr = \int\limits_{0}^{\infty } {} A_{l}^{m} \left( r \right)j_{l} \left( {kr} \right)r^{2} dr = A_{l}^{m\left( l \right)} \left( k \right) $$

where the overhat indicates a spherical Hankel transform and the Hankel transform $A_{l}^{m\left( l \right)} \left( \rho \right)$ is evaluated at $\rho = k$. Hence, Eq. (82) becomes

84 $$ j_{l} \left( {kr_{0} } \right)r_{0}^{2} \frac{d}{dr}\tilde{p}_{l}^{m\;} \left( {r_{0} ,k} \right)\; - \tilde{p}_{l}^{m\;} \left( {r_{0} ,k} \right)r_{0}^{2} \frac{d}{dr}j_{l} \left( {kr_{0} } \right) = - \frac{{ikc_{s} \beta }}{{C_{p} }}\tilde{I}\left( k \right)A_{l}^{m\left( l \right)} \left( k \right) $$
